# Novel Relapsing Fever Spirochete in Bat Tick

**DOI:** 10.3201/eid1403.070766

**Published:** 2008-03

**Authors:** James S. Gill, Amy J. Ullmann, Amanda D. Loftis, Tom G. Schwan, Sandra J. Raffel, Merry E. Schrumpf, Joseph Piesman

**Affiliations:** * Iowa State University, Ames, Iowa, USA; † Centers for Disease Control and Prevention, Fort Collins, Colorado, USA; ‡ Centers for Disease Control and Prevention, Atlanta, Georgia, USA; § Rocky Mountain Laboratories of National Institutes of Health, Hamilton, Montana, USA

**Keywords:** Carios kelleyi, relapsing fever, spirochetes, Borrelia, bat ticks, letter

## Abstract

Novel Relapsing Fever Spirochete in Bat Tick

**To the Editor:** Tick-borne relapsing fever in western North America is a zoonosis caused by spirochetes in the genus *Borrelia* that are transmitted by argasid ticks of the genus *Ornithodoros* (*1*). Human disease occurs in many focal areas and is associated with infections of *Borrelia hermsii*, *B. turicatae*, and possibly *B. parkeri* (*2*,*3*). Although the ecologic parameters that maintain *B. hermsii* and *B. turicatae* differ, human infections usually occur in rustic cabins (*B. hermsii*) and caves (*B. turicata*) inhabited by ticks and their terrestrial vertebrate hosts (*1*). Recently, Gill et al. (*4*) provided evidence that the argasid bat tick, *Carios kelleyi*, feeds upon humans. Subsequently, Loftis et al. (*5*) used PCR analysis and DNA sequencing to detect in *C. kelleyi* an unidentified *Borrelia* species that was closely related to *B. turicatae* and *B. parkeri*. We report the partial molecular characterization of another novel tick-borne relapsing fever spirochete in *C. kelleyi*, which expands our knowledge for this group of pathogenic spirochetes and their potential vertebrate hosts and tick vectors.

*C. kelleyi* were collected August 18, 2005, from a house in Jones County, Iowa, built in 1857. Bats had been excluded from the attic since 1992. Nine months before ticks were collected, bats were prevented from roosting under the eaves. DNA was extracted from 31 nymphal *C. kelleyi*, as described previously (*6*). For each tick, regions of the *glpQ*, *flaB,* and *16S rRNA* genes were amplified and sequenced as described (*3*,*7*,*8*). Sequences were assembled by using the SeqMan program in the Lasergene software package (DNASTAR, Madison, WI, USA).

Fourteen (45.1%) of 31 ticks were positive by PCR for >1 of the genes tested. Partial DNA sequences were determined from tick no. 16, for which amplicons for all 3 genes were obtained. The partial *flaB* sequence had 4 bases different from the 300-base sequence (98.66% identity) reported previously (GenBank accession no. AY763104) for another *Borrelia* sp. found in *C. kelleyi* (*5*). We constructed a 1,992-bp concatenated sequence that contained 1,273 bp of the *16S rRNA*, 351 bp of *flaB*, and 368 bp of *glpQ*. This concatenated sequence was aligned with homologous, trimmed DNA sequences of the same length obtained from representative full-length sequences determined previously for *B. hermsii*, *B. turicatae,* and *B. parkeri* (*3*,*9*) (Figure). This *C. kelleyi* spirochete was more closely related to *B. turicatae* and *B. parkeri* than to *B. hermsii* but was clearly distinct from all 3 species (DNA sequence identities of 98.89%, 98.75%, and 95.98% to *B. turicatae*, *B. parkeri*, and *B. hermsii*, respectively).

**Figure:**
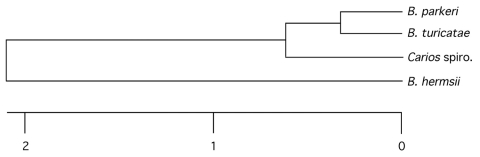
Phylogram comparing the novel spirochete in the bat tick *Carios kelleyi* with *Borrelia parkeri*, *B. turicatae,* and *B. hermsii* based on the concatenated partial *16S rRNA-flaB-glpQ* DNA sequences in the *Carios* spirochete (1,992 bp total) (produced with ClustalV software from DNASTAR [Madison, WI, USA]). Scale bar represents the number of base substitutions per 100 aligned bases. GenBank accession numbers for the *C. kelleyi* spirochete sequences used to construct the tree are EF688575, EF688576, and EF688577. Spiro, spirochete.

A *glpQ* amplicon from another nymphal tick (no. 3) was equenced (GenBank accession no. EF688578) and was unique in the database; it was also considerably different from the *glpQ* sequence determined from tick 16, with 325 of 368 bases matching (88.3% identity). The *Borrelia glpQ* sequence from tick 3 had 85.1%–89.1% identity compared with *glpQ* sequences from *B. hermsii*, *B. turicatae,* and *B. parkeri*. This finding suggests the presence of at least 2 relapsing fever group spirochetes in *C. kelleyi* that await further characterization.

We found a novel *Borrelia* in bat ticks that is closely related to, but distinct from, the other known species of tick-borne relapsing fever spirochetes in North America. The human health implications of the new relapsing fever group spirochete are not yet known. The willingness of *C. kelleyi* to feed on humans and the fact that infection with bacteria closely related to true relapsing fever spirochetes occurs in these ticks suggest that human habitation near bats and their associated tick colonies could pose a public health risk. Growth in laboratory animals or culture could help isolate these novel organisms for further studies to establish the distribution and public health implications of this newly identified *Borrelia* sp.
